# Galectin-3 inhibition reduces fibrotic scarring and promotes functional recovery after spinal cord injury in mice

**DOI:** 10.1186/s13578-024-01310-9

**Published:** 2024-10-15

**Authors:** Fangli Shan, Jianan Ye, Xinzhong Xu, Chao Liang, Yuanzhe Zhao, Jingwen Wang, Fangru Ouyang, Jianjian Li, Jianwei Lv, Zhonghan Wu, Fei Yao, Juehua Jing, Meige Zheng

**Affiliations:** 1 Department of Orthopaedics, The Second Affiliated Hospital of Anhui Medical University, Hefei, 230601 China; 2 Institute of Orthopaedics, Research Center for Translational Medicine, The Second Affiliated Hospital of Anhui Medical University, Hefei, 230601 China; 3 Department of Orthopaedics, Suzhou 100 Hospital, Suzhou, 215000 China

**Keywords:** Spinal cord injury, PDGFRβ, Fibrotic scar, Galectin-3, Macrophage

## Abstract

**Background:**

In the context of spinal cord injury (SCI), infiltrating macrophages assume prominence as the primary inflammatory cells within the lesion core, where the fibrotic scar is predominantly orchestrated by platelet-derived growth factor receptor beta (PDGFRβ^+^) fibroblasts. Galectin-3, a carbohydrate-binding protein of the lectin family, is notably expressed by infiltrating hematogenous macrophages and mediates cell-cell interactions. Although Galectin-3 has been shown to contribute to the endocytic internalization of PDGFRβ in vitro, its specific role in driving fibrotic scar formation after SCI has not been determined.

**Methods:**

We employed a crush mid-thoracic (T10) SCI mouse model. Galectin-3 inhibition after SCI was achieved through intrathecal injection of the Galectin-3 inhibitor TD139 or in situ injection of lentivirus carrying Galectin-3-shRNA (Lv-shLgals3). A fibrosis-induced mice model was established by in situ injection of platelet-derived growth factor D (PDGFD) or recombinant Galectin-3 (rGalectin-3) into the uninjured spinal cord. Galectin-3 internalization experiments were conducted in PDGFRβ^+^ fibroblasts cocultured in conditioned medium in vitro.

**Results:**

We identified the spatial and temporal correlation between macrophage-derived Galectin-3 and PDGFRβ in fibroblasts from 3 to 56 days post-injury (dpi). Administration of TD139 via intrathecal injection or in situ injection of Lv-shLgals3 effectively mitigated fibrotic scar formation and extracellular matrix deposition within the injured spinal cord, leading to better neurological outcomes and function recovery after SCI. Furthermore, the fibrosis-inducing effects of exogenous PDGFD in the uninjured spinal cord could be blocked by TD139. In vitro experiments further demonstrated the ability of PDGFRβ^+^ fibroblasts to internalize Galectin-3, with Galectin-3 inhibition resulting in reduced PDGFRβ expression.

**Conclusions:**

Our finding underscores the pivotal role of macrophage-derived Galectin-3 in modulating the sustained internalized activation of PDGFRβ within fibroblasts, providing a novel mechanistic insight into fibrotic scarring post-SCI.

**Supplementary Information:**

The online version contains supplementary material available at 10.1186/s13578-024-01310-9.

## Introduction

Traumatic spinal cord injury (SCI) disrupts the integrity of the blood-spinal cord barrier, leading to the infiltration of inflammatory cells and the formation of fibrotic scarring at lesion sites [1–3]. Along with infiltrating hematogenous macrophages, perivascular fibroblasts play a significant role in orchestrating these pathological processes [1, 2]. Platelet-derived growth factor receptor beta (PDGFRβ) has been identified as a specific marker for scar-forming fibroblasts [2, 4]. Inhibiting their proliferation has demonstrated promising outcomes in diminishing fibrotic scarring, promoting axonal regeneration, and augmenting locomotor function recovery after SCI [5]. Our recent studies confirmed the pivotal role of platelet-derived growth factor (PDGF)/PDGFRβ pathway activation in fibrotic scar formation [6, 7]. Hematogenous macrophages have been found to recruit fibroblasts to lesion sites, and their depletion reduces fibrotic scar formation and facilitates axonal regrowth [8]. These findings provide valuable insights into the intricate crosstalk mechanism between macrophages and fibroblasts, holding considerable promise for advancing therapeutic strategies in the context of SCI.

Galectin-3, also known as Mac2, is a carbohydrate and N-glycan-binding protein that belongs to the lectin family. While primarily located within the cytoplasm, it can also be secreted onto the cell surface and into the extracellular space [9]. Intracellular Galectin-3 efficiently binds to the highly glycosylated inner membrane, serving as a marker of lysosomal damage and potentially participating in lysosomal repair processes [10, 11]. Galectin-3 has been identified as a long-lasting marker of hematogenous macrophage infiltration into the mouse central nervous system (CNS) after bone marrow transplantation and other CNS diseases [12–14]. After SCI, infiltrating macrophages express high levels of Galectin-3 at the injury site, enabling its use as a specific marker to distinguish hematogenous macrophages from microglia [15, 16]. Galectin-3 knockout mice exhibit an anti-inflammatory phenotype at 7 days post-injury (dpi) and display more preserved fibers at 28 dpi, leading to improved and earlier recovery of locomotor function [17]. Moreover, depletion of Galectin-3^+^ macrophages has been shown to reduce renal fibrosis after unilateral ureteric obstruction [18]. Conversely, exogenous recombinant Galectin-3 significantly increases cardiac fibroblast proliferation and collagen production [19]. The specific mechanism by which Galectin-3 contributes to fibrotic scarring in various fibrotic diseases remains largely unexplored. However, several studies have indicated a relationship between Galectin-3 and PDGFRβ internalization. Extracellular Galectin-3 has been implicated in the formation of distinct endocytic structures and has been demonstrated to mediate the internalization of PDGFRβ in vitro [20, 21]. Despite these findings, the potential role of Galectin-3 in fibrotic scarring after SCI has not been fully elucidated.

In this study, we confirmed the close association between macrophage-derived Galectin-3 and fibroblast-derived PDGFRβ in the lesion core at 3–56 dpi. Inhibiting Galectin-3 resulted in a reduction in fibrotic scar formation and extracellular matrix deposition, promoted neuroprotection, and improved locomotor function recovery after SCI. Furthermore, in situ injection of exogenous PDGFD induced PDGFRβ^+^ fibrosis in the uninjured spinal cord could be blocked by TD139, which is a novel high-affinity inhibitor targeting the carbohydrate binding domain of Galectin-3 and inhibits the recruitment and expansion of macrophages. Macrophage-derived Galectin-3 is internalized by PDGFRβ^+^ fibroblasts in vitro, thereby modulating the expression of PDGFRβ. These results highlight the role of macrophage-derived Galectin-3 in PDGFRβ activation on the surface of fibroblasts, thereby influencing the development of fibrotic scarring after SCI.

## Materials and methods

### Animals

Female C57BL/6J mice aged 8–10 weeks were obtained from the Experimental Animal Center of Anhui Medical University for this study. All experimental procedures were performed in compliance with the Institutional Animal Care Guidelines of Anhui Medical University and approved by the Animal Ethics Committee of Anhui Medical University (Approval No. LLSC20160052). The mice were housed in specific pathogen-free facilities under a 12-h light-dark cycle at a temperature of 24 ± 1 °C with controlled humidity, and had access to food and water.

### Spinal cord Injury Model

The mice were anesthetized with intraperitoneal injection of sodium pentobarbital (50 mg/kg; Harbin Pharmaceutical Group Co., Ltd., Harbin, China) and placed in the prone position on the operating table. The dorsal surface around the T10 segment was shaved, and the skin was disinfected with iodophor. The T9-T11 dorsal skin was dissected, the back muscle was sequentially peeled off. Laminectomy was performed to expose the spinal cord at the T10 level, which served as the clamping site. Crush-induced spinal cord injuries were made using calibrated Dumont #5 forceps (11252-20, Fine Science Tools, Germany) without spacers, with a tip width of 0.5 mm, to completely compress the entire spinal cord laterally from both sides for 5 s [22]. Successful SCI induction was confirmed by observing transient spasms in the hind limbs and tail along with a continuous red clamp mark after saline irrigation. The wound was then sutured layer by layer. Postoperatively, the mice were subjected to auxiliary urination twice daily (morning and afternoon) until spontaneous urination was restored after SCI.

### In situ injection of PDGFD or rGalectin-3

Mice in the experimental group received in situ injection of PDGFD or recombinant Galectin-3 (rGalectin-3) in the uninjured spinal cord. After the T10 spinal cord was exposed as described above, each mouse was placed in a stereotaxic device. The microinjection needle (7634-01 and 7803-05, Hamilton, Switzerland) was inserted 0.3 mm lateral to the midline and 0.8 mm deep into the mouse spinal cord [6]. Two microliters of 100 ng/µl recombinant human PDGFD (1159-SB/CF, R&D Systems, United States) dissolved in 4 mM HCl containing 0.1% BSA or 100 µg/ml rGalectin-3 (HY-P70309, MedChemExpress, United States) dissolved in 100 µl of ddH_2_O was injected into the uninjured spinal cord at a rate of 0.5 µl/min using a stereotaxic injector (KDS LEGATO 130, RWD, China). The control mice received only 2 µl of ddH_2_O. All in situ-injected mice were sacrificed at 7 days post-injection.

### Intrathecal injection of TD139

Prior to injection into the lumbar 5–6 intervertebral space in mice, the dorsal surface was shaved, and the skin was disinfected with iodophor. The successful insertion of the needle into the intradural space was confirmed by sudden tail wagging [22]. Thirty milligrams of TD139 (GC19350, GLPBIO, United States) dissolved in 10 ml PBS (Servicebio, China) containing 3% DMSO (3304, R&D Systems, United States) was injected daily at 1 µl/4 s using a microinjection needle (1701, Hamilton, Switzerland). For the uninjured mice, TD139 was injected immediately after the injection of PDGFD or rGalectin-3 and then injected daily for 7 consecutive days. For the SCI mice, TD139 was injected post-operatively daily until 14 dpi. The control mice received the same volume of 10 µl of PBS containing 3% DMSO.

### In situ injection of lentivirus

The lentiviral vectors utilized in this study were designed by GenePharma (Shanghai, China) and had a virus titer of Lv-shLgals3 at 1.0 × 10E9 TU/ml. The shRNA sequences targeting lgals3 (which encodes Galectin-3) were 5′-ACCCAAACCCTCAAGGATATC-3′ (Lv-shLgals3-144, referred to as Lv-shLgals3 below), 5′-GTAACACGAAGCAGGACAATA-3′ (Lv-shLgals3-657) and 5′-GCTCACCTACTGCAGTACAAC-3′ (Lv-shLgals3-785). A lentivirus expressing a nonspecific shRNA sequence (5′-TTCTCCGAACGTGTCACGT-3′) (Lv-shNC) was employed as a negative control.

The T10 spinal cord crush injury was established according to the experimental methods described above. Subsequently, 1 µl of lentivirus carrying Galectin-3-shRNA (Lv-shLgals3) or empty lentiviral vector (Lv-shNC) was injected in situ at the T10 injury site using a microinjection needle (7634-01 and 7803-05, Hamilton, Switzerland). All mice that received in situ injections of lentivirus were sacrificed at 28 dpi.

### Cell Culture

Mouse mononuclear macrophage leukemia cells (RAW 264.7) were purchased from the Stem Cell Bank, Chinese Academy of Sciences (Shanghai, China) and cultured in complete medium (CM-0190, Procell Life Science & Technology, China). Mouse brain vascular pericytes (referred to as perivascular fibroblasts) were purchased from the National Collection of Authenticated Cell Cultures (Shanghai, China), cultured in complete medium (CM-M222, Procell Life Science & Technology, China), and induced with 250 ng/ml PDGF-BB (220-BB-050, R&D Systems) for 72 h [22]. The cells were cultured in a humidified atmosphere at 37 °C containing 5% CO2.

### Cell treatment

RAW 264.7 macrophages in 24-well plates were transduced with Lv-shNC, Lv-shLgals3-144 (Lv-shLgals3), Lv-shLgals3-657, or Lv-shLgals3-785 at a multiplicity of infection of 100, according to the manufacturer’s protocol (GenePharma). The macrophages were transduced with these lentiviral vectors for 24 h.

For the Galectin-3 internalization experiments, the control group comprised fibroblasts cultured in a 1:1 mixture of fibroblast culture medium and macrophage complete culture medium, and the MP-CM group consisted of fibroblasts cultured with in a 1:1 mixture of fibroblast culture medium and macrophage supernatant conditioned culture medium (MP-CM) for 24 h. The rGalectin-3 group was composed of fibroblasts cultured in medium supplemented with rGalectin-3 (10 µg/ml) for 24 h, and the rGalectin-3 + TD139 group consisted of fibroblasts treated with rGalectin-3 (10 µg/ml) and TD139 (10 µM) for 24 h. The Lv-shNC and Lv-shLgals3 groups were composed of fibroblasts cultured for 24 h with conditioned medium from macrophages transduced with Lv-shNC (10 µl/ml) or Lv-shLgals3 (10 µl/ml), respectively. Immunofluorescence staining of PDGFRβ, Galectin-3, and phalloidin in fibroblasts was performed for the Galectin-3 internalization experiments.

### Histology and immunofluorescence staining

At predetermined time points, each mouse was anesthetized, and cardiac perfusion was performed with cold 0.1 M PBS followed by 4% paraformaldehyde (PFA, Servicebio, China). The tissue near the injury core at the T10 level (3 mm above and below) was removed, fixed in PFA for 5 h, and dehydrated in 30% sucrose solution overnight at 4 °C, embedded in optimum cutting temperature (OCT, BL557A, Biosharp, China) compound, and cut into continuous 16-µm frozen sections with a cryostat (NX50, Thermo Fisher Scientific, United States). The frozen spinal cord sections were washed three times for 5 min each with 0.1 M PBS, blocked in 5% donkey serum in PBS containing 0.3% Triton X-100 (T8200, Solarbio, China) for 1 h at room temperature, and then incubated with primary antibodies in 1% donkey serum containing 0.3% Triton X-100 overnight at 4 °C. The sections were then incubated for 1 h at room temperature with secondary antibodies. The nuclei were stained and sealed with 4,6-diamidino-2-phenylindole (DAPI with an antifluorescence quencher, P0131-25 ml, Beyotime, China) for 5 min. The following primary antibodies were used at the indicated dilutions: goat anti-PDGFRβ (5 µg/ml, AF1042-SP, R&D Systems), rat anti-Galectin-3 (1:200, SC-23938, Santa Cruz Biotechnology), rabbit anti-fibronectin (1:100, 15613-1-AP, Proteintech), rabbit anti-laminin (1:100, 23498-1-AP, Proteintech), rabbit anti-F4/80 (1:100, 28463-1-AP, Proteintech), rabbit anti-neurofilament-heavy (NF-H) (1:400, ab207176, Abcam), rat anti-CD68 (1:300, MCA1957, AbD Serotec), rat anti-GFAP (1:300, 13–0300, Thermo Fisher Scientific), goat anti-5-HT (1:500, #20080, Immunostar), and rabbit anti-NeuN (1:400, ab177487, Abcam). The secondary antibodies used were donkey anti-rat Alexa Fluor 488, donkey anti-rabbit Alexa Fluor 488 (1:500, A-21206, A-21208, Invitrogen), donkey anti-rabbit Alexa Fluor 555, donkey anti-rat Alexa Fluor 555, and donkey anti-goat Alexa Fluor 555 (1:500, A-21432, A-31572, A-48270, Invitrogen). Immunofluorescence images were captured under an Axio Scope A1 microscope (Zeiss, Germany) with a confocal microscope (LSM 900, Zeiss, Germany). Staining colocalization images were created by Zen 3.3 software.

### Immunocytochemistry

The cells were fixed with 4% PFA at room temperature for 10 min, blocked in 5% donkey serum in PBS containing 0.3% Triton X-100 for 1 h at room temperature, and then incubated overnight at 4 °C with primary antibodies in 1% donkey serum containing 0.3% Triton X-100. The cells were then incubated for 1 h at room temperature with secondary antibodies and washed with PBS. The nuclei were stained and sealed with DAPI for 5 min. The following primary antibodies were used at the indicated dilutions: goat anti-PDGFRβ (5 µg/ml, AF1042-SP, R&D Systems) and rat anti-Galectin-3 (1:200, SC-23938, Santa Cruz Biotechnology). The secondary antibodies used included donkey anti-rat Alexa Fluor 555 (1:500, A-48270, Invitrogen), donkey anti-goat Alexa Fluor 647 (1:500, A-21447, Invitrogen), and Actin-Tracker Green-488 (Phalloidin) (1:100, C2201S, Beyotime). Immunofluorescence images were captured under an Axio Scope A1 microscope and a confocal microscope Zen 3.3 software was used to create images of the stained cells.

### Quantitative analysis

Every 5th 16-µm frozen sagittal section that included the entire injured spinal cord was quantified. To evaluate spatiotemporal changes in PDGFRβ^+^ fibroblasts and Galectin-3^+^ macrophages, the maximum range of the positive cells spanning the craniocaudal range at the center of the injury site was quantified for fibroblasts and macrophages at 3–56 dpi [23]. To determine the source of Galectin-3 after SCI, the percentage of Galectin-3^+^ macrophages relative to the total number of F4/80^+^ macrophages at the injured spinal cord was quantified. To quantify PDGFRβ^+^ cell density at injury site after TD139 and Lv-shLgals3 injection, only PDGFRβ^+^ cells that were also DAPI^+^ were counted in the GFAP^−^ region. The number of PDGFRβ^+^ cells in each section was normalized to the area of the GFAP^−^ region. For each mouse, sections including the injury site and two adjacent sagittal sections spaced 160 μm apart were quantified to the area of the GFAP^−^ region, and the counts from at least three sections were averaged [8]. To quantify the area of the fibrotic scar, the immunoreactivities of PDGFRβ, fibronectin, and laminin were normalized through threshold processing, and the areas of the spinal cord segment spanning the lesion site covered by threshold regions were calculated in a 4 × magnification [8]. The immunoreactivity of Galectin-3 or the percentage of the GFAP^−^ area was also normalized through threshold processing to the area of the spinal cord segment spanning the lesion site in a 4 × magnification. The PDGFRβ^+^, fibronectin^+^, laminin^+^, Galectin-3^+^, and GFAP^−^ area were expressed as a percentage of the area of the 2 mm spinal cord segment evaluated. To assess the density of fibers growing at the injury core, threshold regions of the NF-H were measured through threshold processing and according to our previous research [22]. To evaluate axonal regeneration passing through the central core of the injury, the distance from the tip of 5-HT^+^ axons to the GFAP^+^ edge at the rostral end was measured. To assess neuronal preservation, the number of NeuN^+^ cells in the Z1 (0–250 μm), Z2 (250–500 μm), and Z3 (1000–1250 μm) zones adjacent to the lesion core was quantified [24]. The results from each section were averaged with 3–5 samples per group. Image processing was performed using ImageJ/Fiji version 2.3 (NIH, United States). For the in vitro assay, the overall fluorescence intensity of Galectin-3 and PDGFRβ was quantified using ImageJ software after intensity thresholding. The mean fluorescence intensity was calculated for analysis [25].

### Behavioral tests

The Basso Mouse Scale (BMS) was used to evaluate hindlimb motor function after SCI in mice [6]. The walking and limb activity scores of the hindlimbs were observed and recorded according to the protocol developed by Basso and colleagues [26]. Two experienced examiners scored each mouse at 0, 1, 3, 7, 14, 21, and 28 dpi. Gait footprint analysis and motor coordination were assessed at 28 dpi [22]. The forelimb and hind paw were coated with dye of different colors, and the mice were placed on a 4 cm × 80 cm runway covered with white paper. The animals were encouraged to walk straight to the finish line so that representative images of their gaits could be obtained, and stride length, stride width, and paw rotation were assessed to evaluate motor function.

### Statistical analysis

All the data were expressed as the mean ± standard error of the mean (s.e.m). GraphPad Prism 7.0 (GraphPad Software Inc, United States) was used for statistical analysis. One-way analysis of variance (ANOVA), and two-way ANOVA followed by Tukey’s post hoc test were performed to detect differences among multiple groups, or a two-tailed Student’s t test was used to compare two groups. A p value < 0.05 was considered to indicate statistical significance.

## Results

### The spatiotemporal distribution of Galectin-3^+^ macrophages and PDGFRβ^+^ fibroblasts is closely related after SCI

**Fig. 1:**
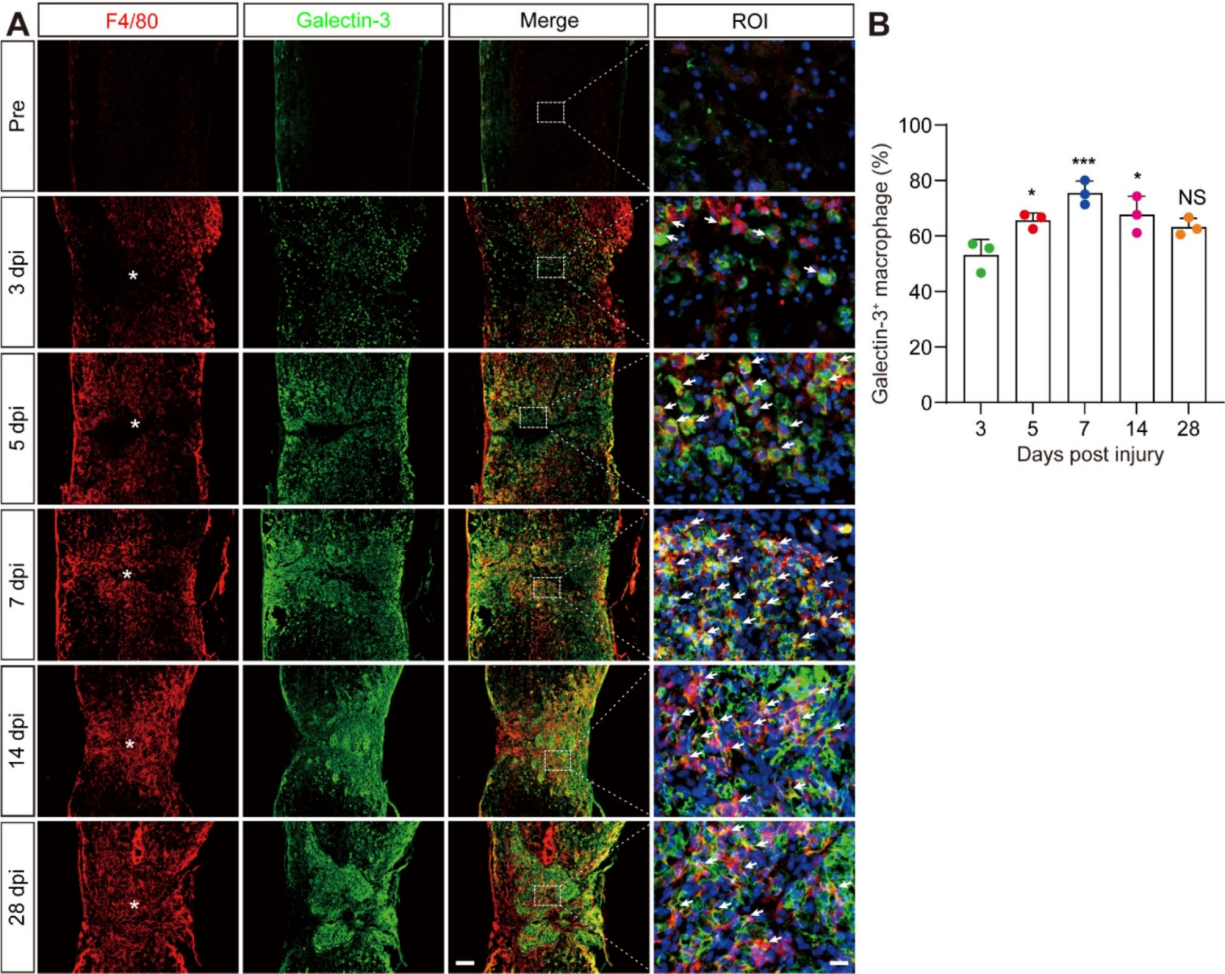
Continuous expression of Galectin-3 in macrophages after SCI. **A** Immunofluorescence staining of F4/80 (red), Galectin-3 (green), and nuclei (blue) in sagittal sections of Pre-injury (Pre) mice and injured mice at 3, 5, 7, 14, and 28 dpi. The region of interest (ROI) represents the dashed boxed region on the left panels. The arrows indicate the colocalization of F4/80 and Galectin-3 observed with a high magnification lens. Asterisks indicate the injured core of the spinal cord. Scale bars: 200 μm (low magnification panel) and 20 μm (high magnification panel). **B** Quantification of the percentage of Galectin-3^+^ macrophages relative to the total number of F4/80^+^ macrophages at the injured spinal cord in (**A**). Data represent mean ± s.e.m. NS, no significance; ^*^P < 0.05 and ^***^P < 0.001 compared with 3 dpi, using one-way ANOVA followed by Tukey’s post hoc test, *n* = 3 per group

**Fig. 2:**
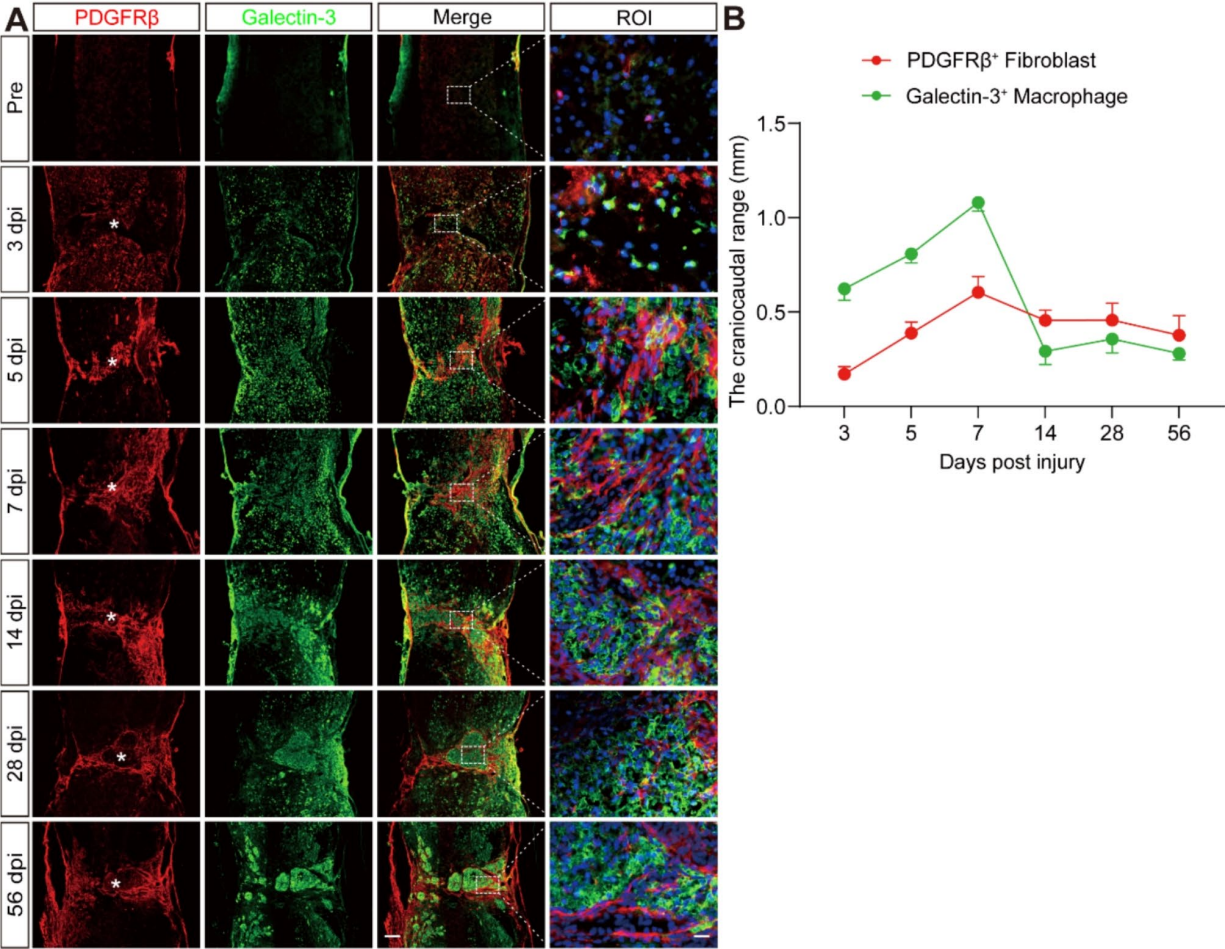
Spatiotemporal distribution of fibroblasts and macrophages after SCI. **A** Immunofluorescence staining of PDGFRβ (red), Galectin-3 (green), and nuclei (blue) in sagittal sections of Pre-injury (Pre) mice and injured mice at 3, 5, 7, 14, 28, and 56 dpi. The ROI represents the dashed boxed region on the left panels. Asterisks indicate the injured core of spinal cord. Scale bars: 200 μm (low magnification panel) and 20 μm (high magnification panel). **B** Quantitative analysis of the craniocaudal range of fibroblasts and macrophages at the lesion site in (**A**). Data represent mean ± s.e.m. *n* = 3 per group

The synergistic colocalization of Galectin-3 with the major macrophage marker F4/80 confirmed the sustained source of Galectin-3 from macrophages at the lesion site post-SCI, as depicted in Fig. 1. To illustrate the crosstalk between Galectin-3^+^ macrophages and PDGFRβ^+^ fibroblasts after SCI, we comprehensively assessed their spatiotemporal distribution through immunofluorescence staining, encompassing both pre-injury and post-injury stages from 3 to 56 dpi. Our findings revealed the absence of Galectin-3 and PDGFRβ expression within the uninjured spinal cord parenchyma (Fig. 2A). In contrast, the lesion site began to exhibit a dispersion of Galectin-3^+^ macrophages and PDGFRβ^+^ fibroblasts as early as 3 dpi, with a notable increase and aggregation at the injury core by 5–7 dpi (Fig. 2A). Over time, the dispersed Galectin-3^+^ macrophages and PDGFRβ^+^ fibroblasts congregated towards the epicenter, with the PDGFRβ^+^ fibroblasts surrounding Galectin-3^+^ macrophages to form a fibrotic scar at 14, 28, and 56 dpi (Fig. 2A-B). Collectively, these results illustrate the prolonged coexistence of macrophage-derived Galectin-3 with the fibrotic scar inducing factor PDGFRβ after SCI, suggesting a potential interactive relationship between the two molecules.

### Blocking Galectin-3 reduces the extent of fibrotic scarring, improves neurological outcomes and motor function recovery after SCI

**Fig. 3:**
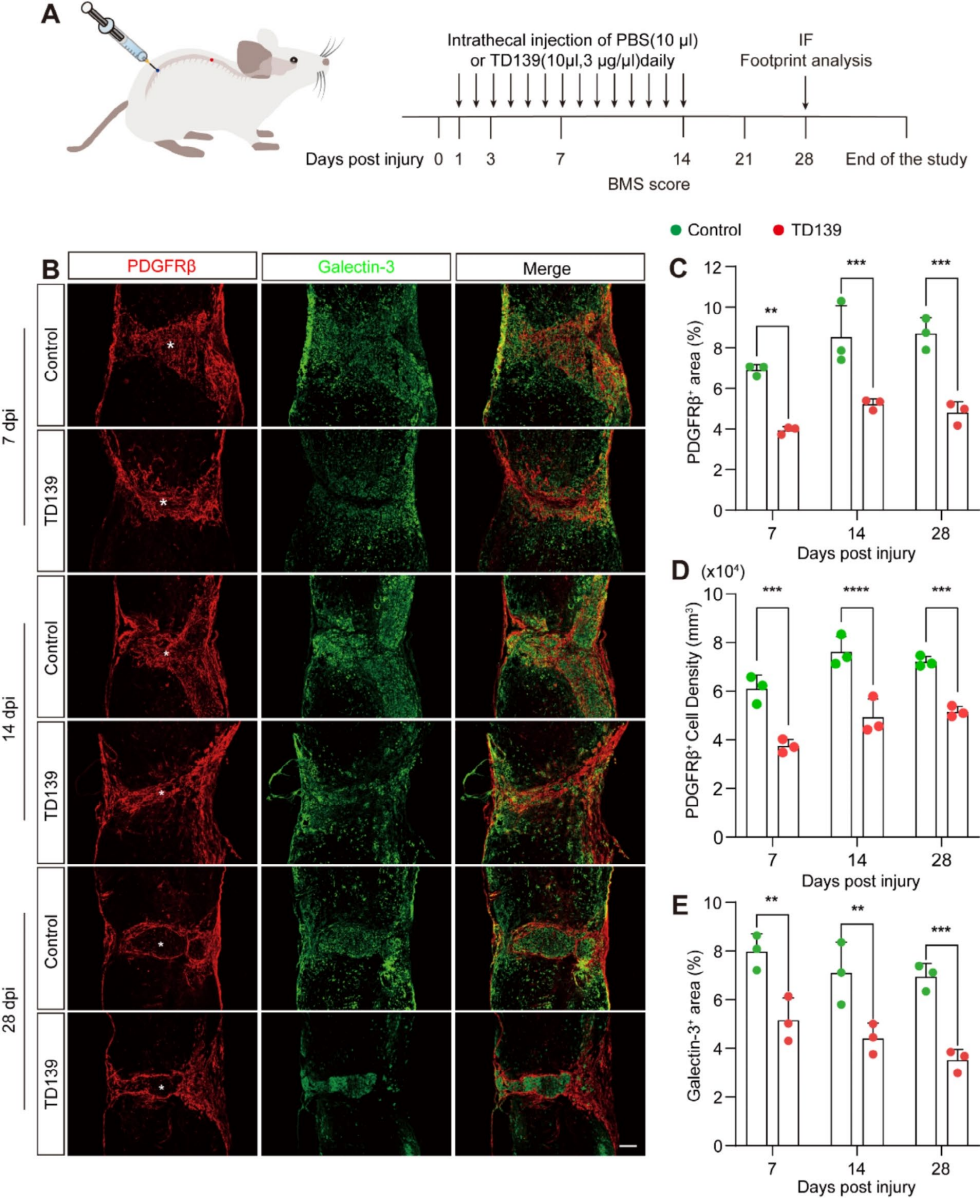
Reduction in fibrotic scarring through intrathecal injection of the Galectin-3 inhibitor TD139 after SCI. **A** Experimental setup scheme. The control (10 µl of PBS containing 3% DMSO) or TD139 (10 µl, 3 µg/µl) was intrathecally injected daily from 1 to 14 dpi. **B** Immunofluorescence staining of PDGFRβ (red) and Galectin-3 (green) in sagittal sections of the control and TD139 groups at 7, 14, and 28 dpi. Asterisks indicate the injured core of the spinal cord. Scale bar: 200 μm. **C** Quantification of the percentage of the PDGFRβ^+^ area within the spinal cord segment spanning the injured core in (**B**). **D** Quantification of the density of PDGFRβ^+^ cell within the spinal cord segment spanning the injured core in (**B**). **E** Quantification of the percentage of the Galectin-3^+^ area within the spinal cord segment spanning the injured core in (**B**). Data represent mean ± s.e.m. ^**^P < 0.01, ^***^P < 0.001, and ^****^P < 0.0001 using two-way ANOVA followed by Tukey’s post hoc test, *n* = 3 per group

To investigate the impact of Galectin-3 on fibrotic scarring, a Galectin-3 inhibitor (TD139) was intrathecally administered daily until 14 dpi (Fig. 3A). Immunofluorescence staining revealed that, compared with the control group, the density of PDGFRβ^+^ cells, as well as the PDGFRβ^+^ and Galectin-3^+^ areas, were significantly reduced at 7, 14, and 28 dpi following TD139 treatment (Fig. 3B-E). This indicates that TD139 effectively inhibited fibrotic scar formation and macrophage infiltration. Furthermore, the fibronectin^+^ and laminin^+^ immunofluorescence areas at the lesion core were significantly lower in the TD139 group than those in the control group (Fig S1), suggesting a concomitant decrease in fibroblast-related extracellular matrix deposition after SCI. Therefore, our results indicate that inhibiting Galectin-3 aggregation at an early stage can lead to chronic reduction of fibrotic scarring at the lesion core.

**Fig. 4:**
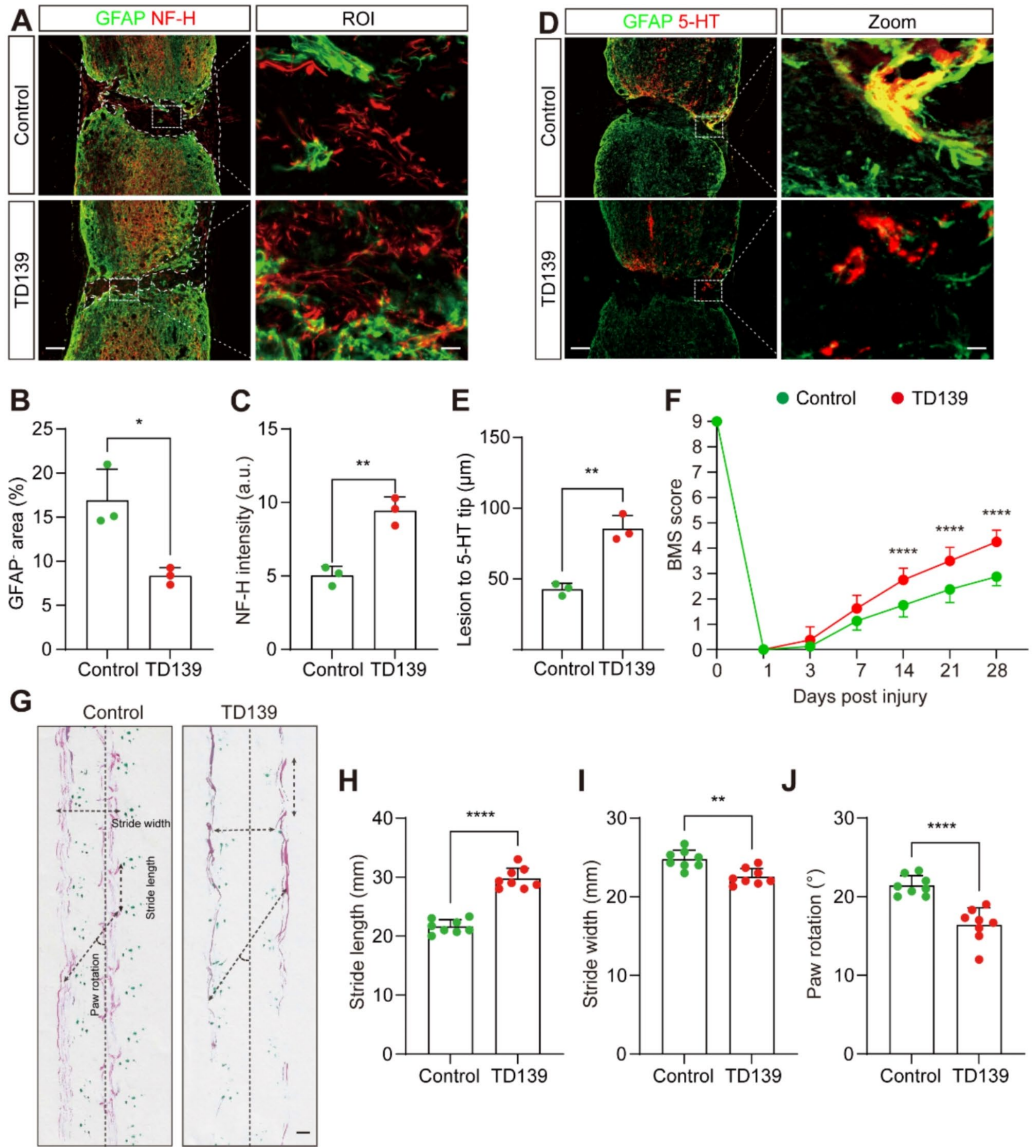
Pharmacological inhibition of Galectin-3 facilitates neuroprotection and functional recovery after SCI. **A** Immunofluorescence staining of GFAP (green) and NF-H (red) in sagittal sections of the control and TD139 groups at 28 dpi. The ROI represents the dashed boxed region on the left panels. Scale bars: 200 μm (low magnification panel) and 20 μm (high magnification panel). **B** Quantification of the percentage of GFAP^−^ area indicated by the white dashed lines at the injury site in (**A**). **C** Quantification of NF-H intensity at the injury site in (**A**). **D** Immunofluorescence staining of GFAP (green) and 5-HT (red) in sagittal sections of the control and TD139 groups at 28 dpi. The Zoom represents the dashed boxed region on the left panels. Scale bars: 200 μm (low magnification panel) and 20 μm (high magnification panel). **E** Quantification of the lesion distance to the 5-HT axon tip at the injury site in (**D**). **F** The BMS score was determined at the indicated time points. **G** Footprint assays of mice in the control and TD139 groups at 28 dpi. The dotted lines represented the direction of walking and parameter measurements were shown in footprint assays. Scale bars: 10 mm. **H-J** Quantification of stride length (**H**), stride width (**I**), and paw rotation (**J**) at 28 dpi. Data represent mean ± s.e.m. ^*^P < 0.05, ^**^P < 0.01, and ^****^P < 0.0001 by unpaired two-tailed Student’s t test (**B**, **C**, **E**, **H**, **I**, and **J**). ^****^P < 0.0001 by two-way ANOVA followed by Tukey’s post hoc test (**F**). *n* = 3 per group (**B**, **C** and **E**), *n* = 8 per group (**F**, **H**, **I**, and **J**)

We further assess the therapeutic effect of Galectin-3 blockade on tissue preservation and repair following SCI. Immunofluorescence staining showed a significant increase in NF-H^+^ axon density within the lesion core at 28 dpi in the TD139 group compared with the control group, accompanied by a pronounced reduction in lesion size indicated by GFAP^−^ area (Fig. 4A-C). Moreover, in the TD139 group, there were obviously more 5-HT^+^ axon fibers crossing the lesion core compared to the control group at 28 dpi (Fig. 4D-E). In addition, the TD139-treated mice displayed improved hindlimb coordination and better locomotor function recovery compared to controls, as determined by BMS motor function scores and footprint analysis (Fig. 4F-J). These findings demonstrate that pharmacological inhibition of Galectin-3 after SCI mitigates fibrotic scarring and facilitates neuroprotection, leading to better neurological outcomes and motor function recovery.

### Lentiviral-mediated Galectin-3 gene silencing attenuates fibrotic scarring and enhances neurological recovery post-SCI

**Fig. 5:**
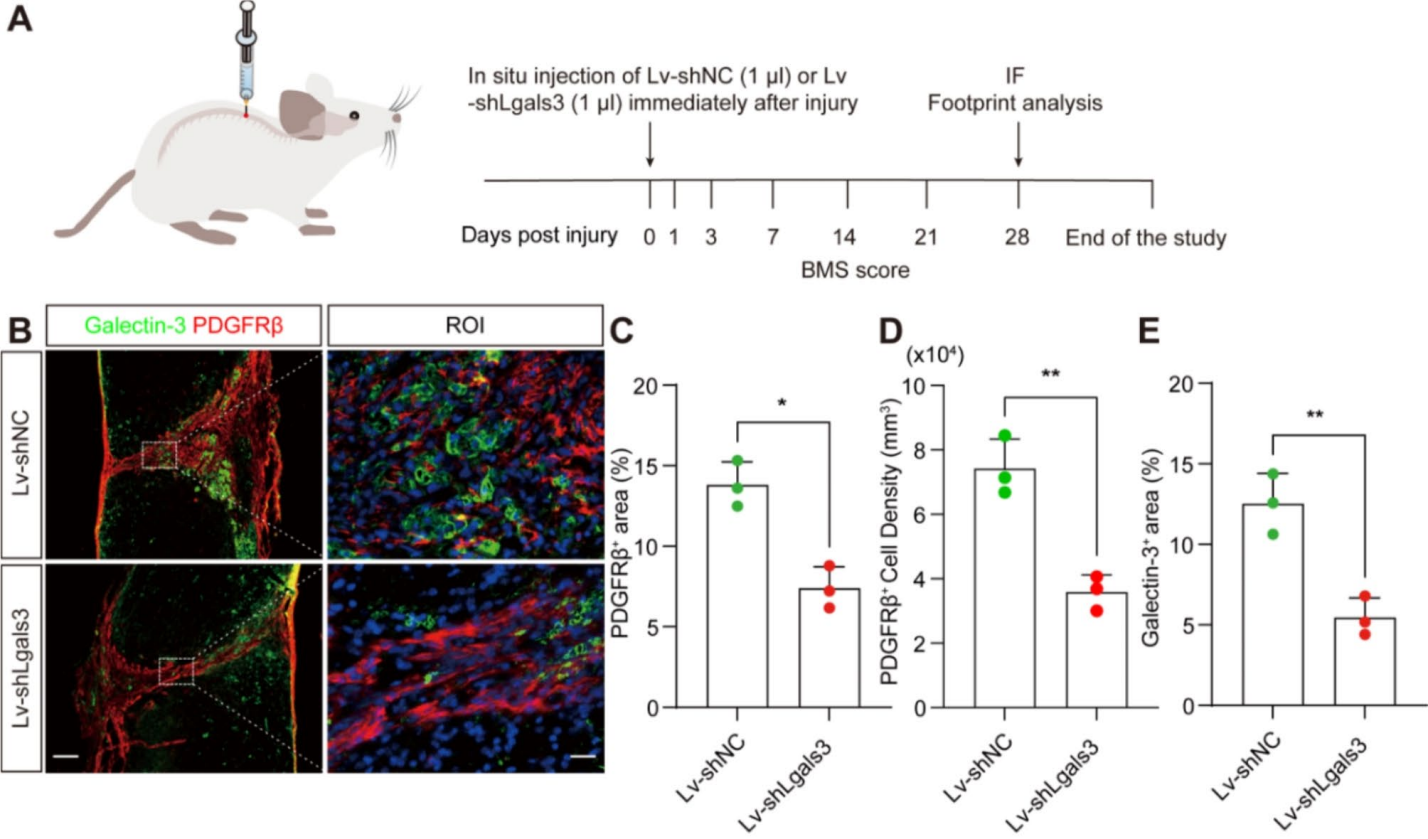
In situ injection of Lv-shLgals3 reduces fibrotic scarring after SCI. **A** Experimental setup scheme. In situ injection of Lv-shNC (1 µl) or Lv-shLgals3 (1 µl) immediately after SCI. **B** Immunofluorescence staining of PDGFRβ (red) and Galectin-3 (green) in sagittal sections of the Lv-shNC and Lv-shLgals3 groups at 28 dpi. The ROI represents the dashed boxed region on the left panels. Scale bars: 200 μm (low magnification panel) and 20 μm (high magnification panel). **C** Quantification of the percentage of the PDGFRβ^+^ area within the spinal cord segment spanning the injured core at 28 dpi. **D** Quantification the density of PDGFRβ^+^ cell within the spinal cord segment spanning the injured core at 28 dpi. **E** Quantification of the percentage of the Galectin-3^+^ area within the spinal cord segment spanning the injured core at 28 dpi. Data represent mean ± s.e.m. ^*^P < 0.05 and ^**^P < 0.01 by unpaired two-tailed Student’s t test, *n* = 3 per group

Since TD139 is a novel high-affinity inhibitor targeting the Galectin-3 carbohydrate binding domain, through the inhibition of recruitment and expansion of Galectin-3-secreting macrophages [27, 28], we constructed a lentivirus to directly knock down the expression of Galectin-3, aiming to corroborate its role in modulating fibrotic scar formation, neurological prognosis, and motor function restoration following SCI. The lentivirus (Lv-shLgals3) was administered in situ to the lesion epicenter for targeted Galactin-3 gene knockdown (Fig. 5A). Comprehensive validation of lentivirus efficacy is presented in the supplementary material (Fig S2, Fig S3, and Fig S4). Notably, we observed a significant decrease in Galectin-3^+^CD68^+^ macrophages at 28 dpi in the injured spine cord after Lv-shLgals3 injection, suggesting that the lentivirus effectively knocked down Galectin-3 expression, thereby suppressing the inflammatory response (Fig S4). Subsequent immunofluorescence staining demonstrated a significant reduction in the areas positive for PDGFRβ and Galectin-3, as well as a decrease in PDGFRβ^+^ cell density, at 28 dpi post Lv-shLgals3 administration, in contrast to the Lv-shNC control group (Fig. 5B-E). These observations indicate that lentiviral-mediated gene silencing may surpass Galectin-3 inhibition of TD139 in mitigating fibrotic scar formation, suggesting a more pronounced and direct effect when Galectin-3 expression is suppressed.

**Fig. 6:**
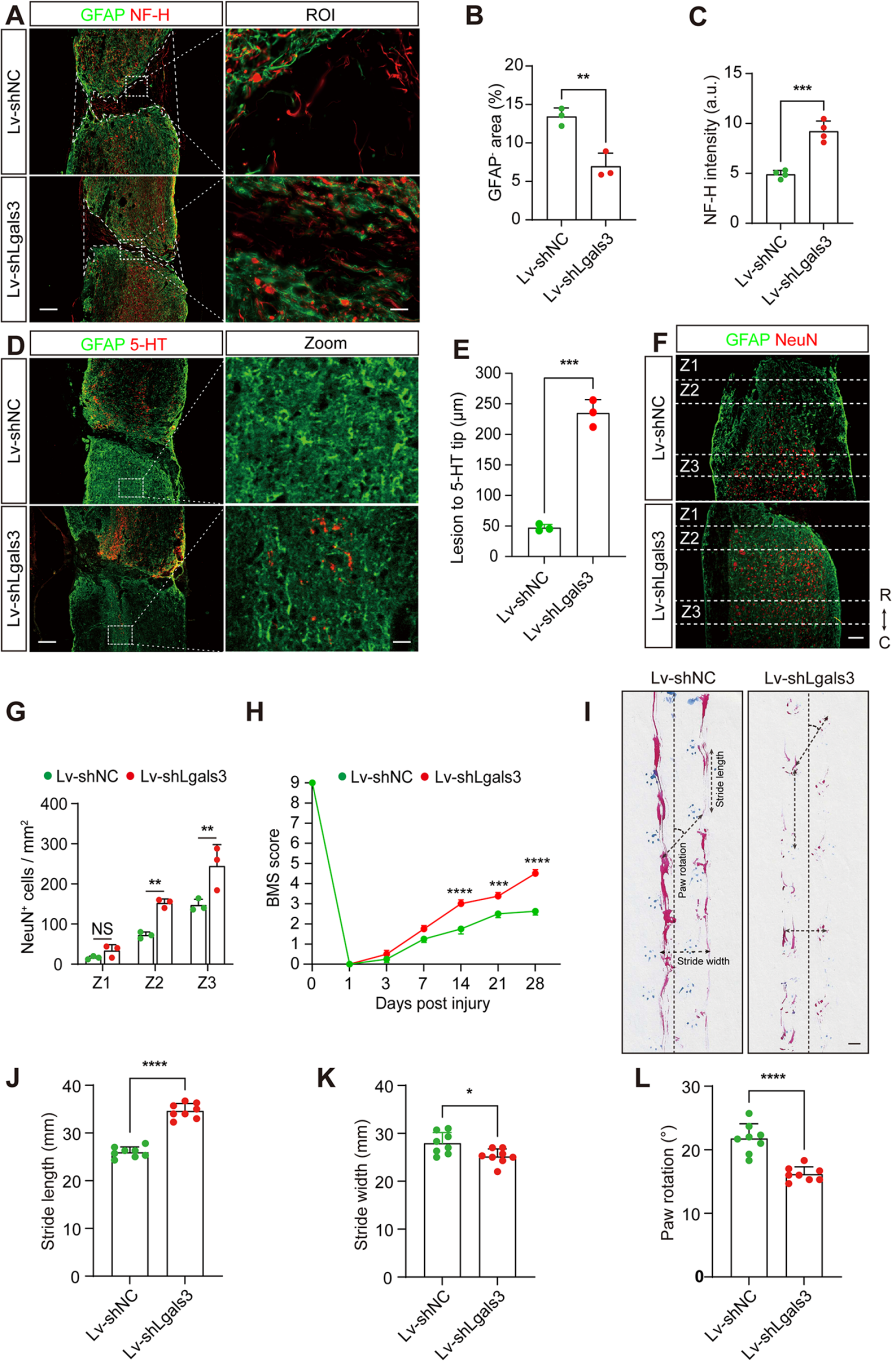
In situ injection of Lv-shLgals3 promotes wound healing, neuroprotection, and functional recovery after SCI. **A** Immunofluorescence staining of GFAP (green) and NF-H (red) in sagittal sections of the Lv-shNC and Lv-shLgals3 groups at 28 dpi. The ROI represents the boxed region on the left panels. Scale bars: 200 μm (low magnification panel) and 20 μm (high magnification panel). **B** Quantification of the percentage of GFAP^−^ area indicated by the white dashed lines at the injury site in (**A**). **C** Quantification of NF-H intensity at the injury site in (**A**). **D** Immunofluorescence staining of GFAP (green) and 5-HT (red) in sagittal sections of the Lv-shNC and Lv-shLgals3 groups at 28 dpi. The Zoom represents the dashed boxed region on the left panels. Scale bars: 200 μm (low magnification panel) and 20 μm (high magnification panel). **E** Quantification of the lesion distance to the 5-HT axon tip at the injury site in (**D**). **F** Immunofluorescence staining of GFAP (green) and NeuN (red) in sagittal sections of the Lv-shNC and Lv-shLgals3 groups at 28 dpi. Scale bar: 200 μm. **G** Quantification of the number of NeuN^+^ cells in the Z1-Z3 zones adjacent to the lesion core in (**F**). **H** The BMS score was determined at the indicated time points. **I** Footprint assays of mice in the Lv-shNC or Lv-shLgals3 groups at 28 dpi. The dotted lines represented the direction of walking and parameter measurements were shown in footprint assays. Scale bars: 10 mm. **J-L** Quantification of stride length (**J**), stride width (**K**), and paw rotation (**L**) at 28 dpi. Data represent mean ± s.e.m. ^*^P < 0.05, ^**^P < 0.01, ^***^P < 0.001, and ^****^P < 0.0001 by unpaired two-tailed Student’s t test (**B**, **C**, **E**, **J**, **K**, and **L**). NS, no significance; ^**^P < 0.01 and ^****^P < 0.0001 by two-way ANOVA followed by Tukey’s post hoc test (**G** and **H**). *n* = 3 or 4 per group (**B**, **C**, **E**, and **G**), *n* = 8 per group (**H**, **J**, **K**, and **L**)

We also examined the impact of lentiviral Galectin-3 knockdown on neurological outcomes and motor function recovery. The Lv-shLgals3 in situ injection group exhibited improvements in NF-H^+^ axon density and GFAP^−^ area comparable to those observed in the TD139 group (Fig. 6A-C). Furthermore, there were distinctly more 5-HT^+^ axon fibers crossing the injured site in the Lv-shLgals3 group compared to the Lv-shNC group at 28 dpi (Fig. 6D-E). Additionally, the quantity of NeuN^+^ cells in the Z2-Z3 zones adjacent to the lesion core was significantly higher in the Lv-shLgals3 group than in the Lv-shNC group at 28 dpi (Fig. 6F-G). Mice in the Lv-shNC group exhibited markedly inferior recovery compared to the Lv-shLgals3 group at 14, 21, and 28 dpi (Fig. 6H). Footprint analysis revealed that the Lv-shLgals3-treated SCI mice maintained more consistent hindlimb coordination (evidenced by red ink) relative to Lv-shNC-treated SCI mice at 28 dpi, as denoted by enhanced stride length, reduced stride width and diminished paw rotation (Fig. 6I-L). Overall, these findings affirm the comparable efficacy of lentiviral interventions in fostering neurologic prognosis and motor function recuperation after SCI.

### Inhibition of Galectin-3 blocks PDGFD-induced fibrosis in the uninjured spinal cord

**Fig. 7:**
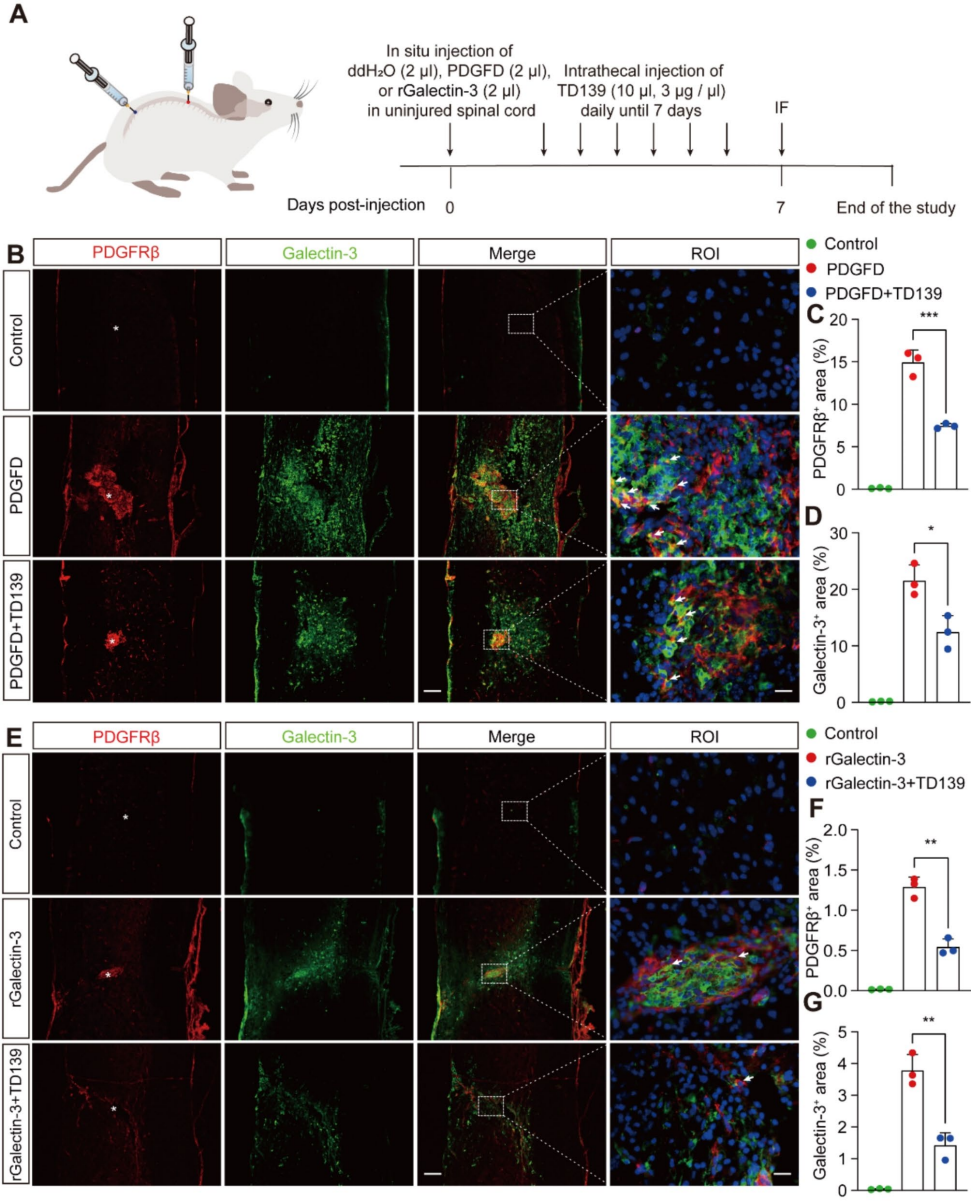
Inhibition of Galectin-3 effectively prevents PDGFD-induced PDGFRβ^+^fibrosis in the uninjured spinal cord. **A** Scheme of the experimental setup. Following in situ injection of control (2 µl ddH_2_O), PDGFD (2 µl), or rGalectin-3 (2 µl) into the uninjured spinal cord, TD139 (10 µl, 3 µg/µl) was intrathecally injected daily from 0 to 7 days post-injection. **B** Immunofluorescence staining of PDGFRβ (red) and Galectin-3 (green) in sagittal sections of the control, PDGFD, and PDGFD + TD139 groups at 7 days post-injection. The ROI represents the dashed boxed region on the left panels. The arrows indicate the colocalization of Galectin-3 and PDGFRβ observed with a high magnification lens. Asterisks indicate the injection sites in the spinal cord. Scale bars: 200 μm (low magnification panel) and 20 μm (high magnification panel). **C-D** Quantification of the percentage of PDGFRβ^+^ area (**C**) or Galectin-3^+^ area (**D**) within the spinal cord segment surrounding the injection site in (**B**). **E** Immunofluorescence staining of the PDGFRβ (red) and Galectin-3 (green) in sagittal sections of the control, rGalectin-3, and rGalectin-3 + TD139 groups at 7 days post-injection. **F-G** Quantification of the percentage of the PDGFRβ^+^ area (**F**) or Galectin-3^+^ area (**G**) within the spinal cord segment surrounding the injection site in (**E**). Data represent mean ± s.e.m. ^*^P < 0.05, ^**^P < 0.01, and ^***^P < 0.001 (**C**, **D**, **F**, and **G**) by one-way ANOVA followed by Tukey’s post hoc test, *n* = 3 per group

Our recent investigations have demonstrated that exogenous PDGFD injection into the uninjured spinal cord precipitates PDGFD/PDGFRβ pathway activation, leading to fibrotic outcomes [6]. To elucidate Galectin-3’s direct role in modulating the PDGFD/PDGFRβ pathway in vivo and its regulatory influence on PDGFRβ-associated fibrosis, we established a PDGFD or rGalectin-3 in situ injection model (Fig. 7A). Consistent with prior findings, PDGFD administration induced robust PDGFRβ^+^ fibrosis accompanied by substantial Galectin-3 expression at the fibrotic site (Fig. 7B-D). Notably, the fibrotic response elicited by PDGFD was significantly abrogated following TD139 intrathecal administration, as evidenced in the PDGFD + TD139 group (Fig. 7B-C). To ascertain whether Galectin-3 alone, or in combination with TD139, could directly trigger fibrosis, we administrated Galectin-3 in situ either alone or in conjunction with daily TD139 intrathecal injection (Fig. 7E). The findings indicated that Galectin-3 alone induced minimal fibrosis (approximately 1.3%), markedly less than that provoked by PDGFD (approximately 15%), and that concurrent TD139 administration effectively blocked Galectin-3-induced fibrosis (Fig. 7E-G). These results suggest that while Galectin-3 may activate or potentiate PDGFRβ, its effects are substantially less pronounced than those of the specific ligand, PDGF. Additionally, we observed partial colocalization of PDGFRβ and Galectin-3 as shown in Fig. 7B and E. Taken together, these data confirm that Galectin-3 is essential for the activation of PDGFD/PDGFRβ pathway during fibrosis and demonstrate that Galectin-3 inhibition reduces PDGFD-induced PDGFRβ^+^ fibrosis.

### Galectin-3 internalization by PDGFRβ^+^ fibroblast promotes PDGFRβ expression in vitro

**Fig. 8:**
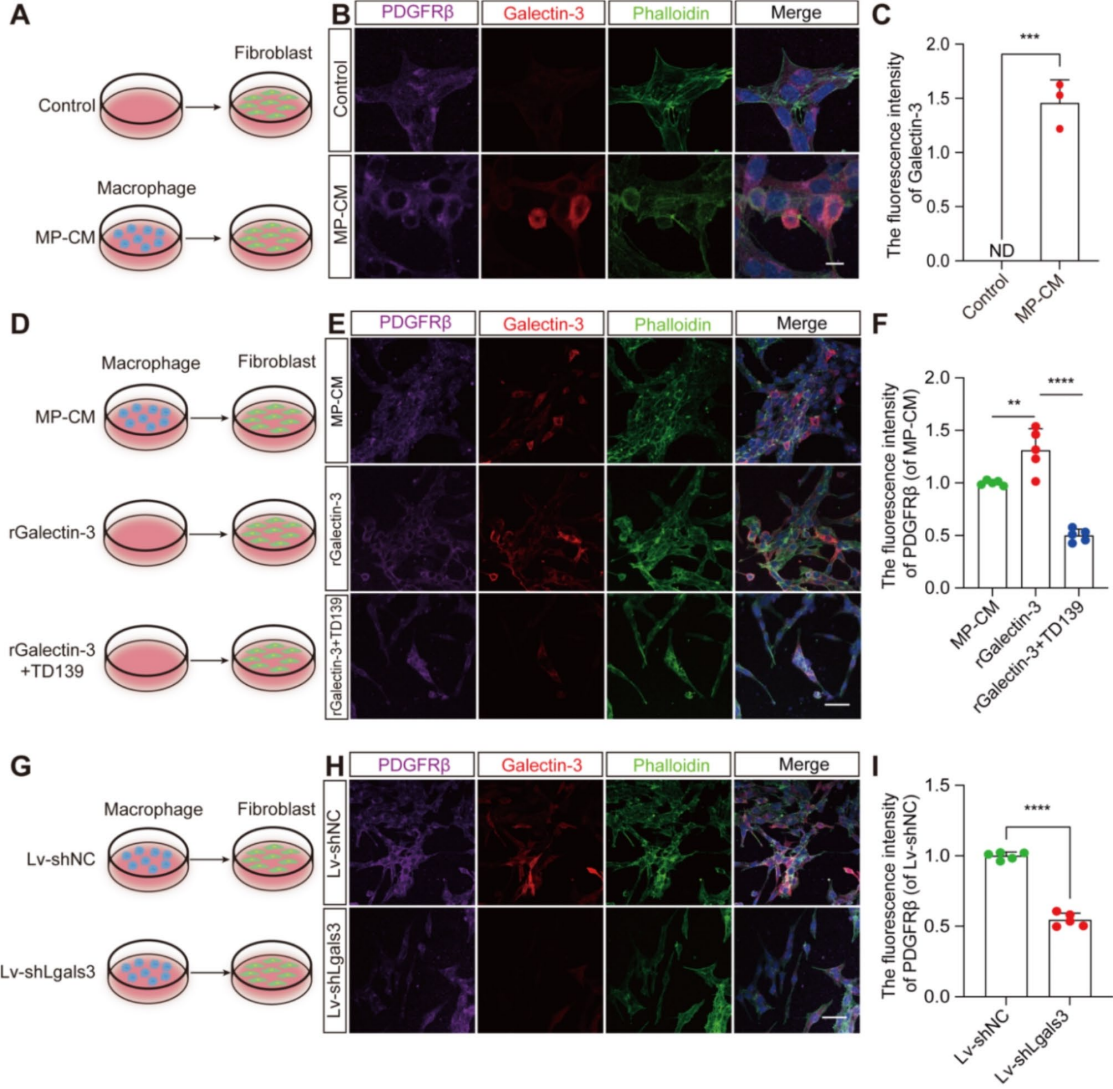
Macrophage-derived Galectin-3 is internalized by PDGFRβ^+^ fibroblasts and blocking it reduces PDGFRβ expression in vitro. **A** Schematic diagram of culture experiment with fibroblasts in normal culture medium (control) or macrophage supernatant conditioned culture medium (MP-CM) for 24 h. **B** Immunofluorescence staining of PDGFRβ (purple), Galectin-3 (red), and phalloidin (green) in fibroblasts from the control and MP-CM groups. Scale bar: 10 μm. **C** Quantification of the fluorescence intensity of Galectin-3 in (**B**). **D** Schematic diagram of fibroblast culture media supplemented with different conditioned media containing MP-CM, rGalectin-3, or rGalectin-3 + TD139 for 24 h. **E** Immunofluorescence staining of PDGFRβ (purple), Galectin-3 (red), and phalloidin (green) in fibroblasts from the MP-CM, rGalectin-3, and rGalectin-3 + TD139 groups. Scale bar: 50 μm. **F** Quantification of the fluorescence intensity of PDGFRβ in (**E**). **G** Schematic diagram of the culture experiment with fibroblasts in conditioned medium from macrophages transduced with Lv-shNC or Lv-shLgals3 for 24 h. **H** Immunofluorescence staining of PDGFRβ (purple), Galectin-3 (red), and phalloidin (green) in fibroblasts from the Lv-shNC and Lv-shLgals3 groups. Scale bar: 50 μm. **I** Quantification of the fluorescence intensity of PDGFRβ in (**H**). The nuclei are stained blue with DAPI. Data represent mean ± s.e.m. ^***^P < 0.001 and ^****^P < 0.0001 by unpaired two-tailed Student’s t test (**C** and **I**). ND, not determined; ^**^P < 0.01 and ^****^P < 0.0001 by one-way ANOVA followed by Tukey’s post hoc test (**F**). *n* = 3 or 5 per group

Galectin-3 has been implicated in mediating PDGFRβ receptor internalization in vitro [20, 21]. To further probe the molecular mechanisms underlying Galectin-3-mediated fibroblast-associated fibrosis, we investigated whether Galectin-3 could be endocytosed by PDGFRβ^+^ fibroblasts in vitro (Fig. 8A). Co-culturing perivascular fibroblasts with macrophage-conditioned medium for 24 h revealed that macrophage-secreted Galectin-3 was internalized within PDGFRβ^+^ fibroblasts, as evidenced by triple-labeled staining for PDGFRβ, Galectin-3, and phalloidin (Fig. 8B-C). We also evaluated the uptake of exogenous rGalectin-3 by fibroblasts and the effect of specific Galectin-3 blockade on PDGFRβ expression (Fig. 8D-F). Our results demonstrated that exogenous rGalectin-3 is internalized by PDGFRβ^+^ fibroblasts and that blocking Galectin-3 led to diminished PDGFRβ expression (Fig. 8E-F). These findings indicate that Galectin-3 uptake by fibroblasts promotes the expression of PDGFRβ. Further assessment of PDGFRβ expression in fibroblasts exposed to conditioned medium from macrophages transduced with Lv-shNC or Lv-shLgals3 (Fig. 8G) revealed that disruption of macrophage-derived Galectin-3 via Lv-shLgals3 downregulated PDGFRβ expression in fibroblasts (Fig. 8H-I). Collectively, these insights suggest a pivotal role for Galectin-3 in enhancing PDGFRβ expression within fibroblasts through a mechanism involving its internalization.

## Discussion

After SCI, macrophage aggregation and PDGFRβ-driven fibrotic scarring together impede axonal regeneration, but their synergistic mechanisms remain unclear [8]. Our investigation sheds light on the spatial and temporal correlation between macrophage-derived Galectin-3 and PDGFRβ in fibroblasts. We demonstrate that targeted inhibition of Galectin-3, either through intrathecal administration of TD139 or via in situ delivery of Lv-shLgals3, mitigates fibrotic scar formation, leading to enhanced neurological outcomes and motor function recovery after SCI. Inhibition of Galectin-3 remarkably blocks PDGFD-induced fibrosis in the uninjured spinal cord. Furthermore, our in vitro data reveal that macrophage-derived Galectin-3 can be internalized by PDGFRβ^+^ fibroblasts, potentially upregulating PDGFRβ expression. These findings underscore the vital role of Galectin-3 in modulating PDGFRβ-related fibrosis after SCI.

The work of Y Zhu et al., employing lysM^tdTom^> Cx3cr1^GFP^ chimeric mice and col1α1^GFP^ mice models of SCI, has provided valuable insights into the intricate interplay between hematogenous macrophages and fibroblasts within the fibrotic scar, highlighting the essential role of macrophages in both fibrotic scar formation and axonal regeneration. Their studies reveal that depletion of macrophages rather than microglia, which are limited to the surrounding astrocytic scar, leads to a decrease in fibroblast density and, consequently, increased axonal growth within the fibrotic scar [8]. This is accompanied by diminished expression of a proliferation inducing ligand (APRIL), with APRIL knockout mice exhibiting reduced fibrotic scarring and improved axonal regeneration, potentially due to decreased macrophage infiltration after SCI [29]. Kazu Kobayakawa et al., have further unraveled the mechanism by which infiltrating macrophages migrate to the injury core, process dependent on the gradient of complement component C5a, mainly expressed by PDGFRβ^+^ fibroblasts [30]. Macrophages have been reported to contribute to wound compaction and recovery through Plexin-B2 after SCI [31]. Clodronate-induced macrophage apoptosis within 14 dpi not only results in the loss of this wound-compacting function, but may also exacerbate the extent of the injury, leading to a temporary expansion of the GFAP^−^ area during the period of clodronate treatment [8]. In contrast, our treatment with TD139 or Lv-shLgals3 does not induce macrophage apoptosis. Instead, it inhibits PDGFRβ pathway activation and reduces macrophage recruitment, leading to a favorable outcome of simultaneous reduction in both fibrotic scarring and the GFAP^−^ area. The glial scar boundary, composed of proliferating astrocytes organized via signal transducer and activator of transcription 3 (STAT3)-dependent mechanisms, corrals inflammatory and fibrotic cells into discrete areas [24]. A limitation of our study is that we have not yet confirmed whether targeting Galectin-3 with TD139 or Lv-shLgals3 alters astrocyte phenotypes or affects key downstream signaling factors like STAT3, which could influence the role of astrocytes in promoting tissue repair.

Galectin-3, a multifaceted protein predominantly emanating from monocytes/ macrophages, contains a carbohydrate binding domain and plays a pro-inflammatory role in various diseases [32, 33]. Intracellularly, Galectin-3 binds with N-acetyllactosamine and colocalizes in damaged lysosomes [10]. Jingyue Jia et al. have shown that Galectin-3 recruits endosomal sorting complexes required for transport (ESCRT) components to damaged lysosomes, facilitating their repair and functional restoration [11]. As a monomer, extracellular Galectin-3 is recruited to membranes by binding to N-glycosylated cargo proteins, subsequently aggregating into pentameric structures that are crucial for the uptake of clathrin-independent carriers (CLICs) cargo [21]. Madison A Rogers et al. have posited that the PDGFRβ receptor can be internalized through the CLICs pathway [34], suggesting a role for Galectin-3 in PDGFRβ internalization. Beyond receptor internalization, extracellular Galectin-3 is implicated in the pathogenesis of diverse diseases, modulating fundamental cellular functions including cell-cell, and cell-matrix interactions, as well as inflammation [35].

Prior research on Galectin-3 has mainly focused on its role in the inflammatory response. Evidence indicates that extracellular interactions between Galectin-3 and toll-like receptor 4 (TLR4) can modulate the inflammatory cascade, with neutralizing antibody against Galectin-3 capable of attenuating lipopolysaccharide-induced inflammation [36]. Moreover, the upregulation of Galectin-3 in damaged lysosomes has been linked to inflammation through pathways dependent on NFκB and NLRP3 inflammasome in Huntingtin’s disease [37]. In CNS diseases, Galectin-3 serves as a specific histological marker to distinguish peripherally derived myeloid cells, primarily monocytes/macrophages, from resident microglia under both homeostatic and neuroinflammatory conditions [13]. Given its persistent expression at the injury site, as indicated in this study, we highlight that Galectin-3 plays an important role in the pathological processes after SCI.

The inflammatory response is one of the key factors influencing fibrotic scar formation in various organ injuries. Emerging research has consistently shown that Galectin-3 mediates fibrosis, exemplified by inhibiting Galectin-3 that reduces the inflammatory activation of TLR4 and downstream NF-κB to prevent myocardial fibrosis [38]. Furthermore, the downregulation of Galectin-3 transcription has been associated with a reduction in inflammatory cell polarization and a decreased secretion of the profibrotic factors such as Arg-1, CD206, IL-10, and TGF-β, resulting in diminished renal fibrosis [39]. These studies underscore the significance of Galectin-3 in the fibrotic processes affecting various organs and tissues. The sustained expression of Galectin-3 at the injury site after SCI not only suggests a lysosomal dysfunction in macrophages leading to formation of foamy cells and triggering an inflammatory state but may also be implicated in the regulation of fibrotic scar formation by fibroblasts. Given Galectin-3’s dual role in mediating inflammation and glycosylation receptor endocytosis activation, we propose that targeting Galectin-3 with TD139 or Lv-shLgals3 could reduce macrophage infiltration and attenuate the inflammatory response, as well as block Galectin-3-mediated PDGFRβ activation, thereby effectively reducing fibrotic scar formation. However, the relationship between inflammation and fibrosis is complex, and the precise mechanisms underlying this process remain to be elucidated. Therefore, our study specifically aims to investigate the direct effects of Galectin-3 on the PDGFRβ pathway in fibrosis scarring.

The PDGFRβ receptor, activated by PDGF, is known to drive crucial cellular processes such as proliferation, migration and extracellular matrix production [40, 41]. Previous investigations have demonstrated that Galectin-3 could be induced by PDGF in pulmonary arterial smooth muscle cells (PASMCs), and mediates the effect of PDGF on PASMC proliferation and migration in pulmonary arterial hypertension [42]. Our research has established that the PDGF/PDGFRβ signaling pathway is critical for mediating the pericyte-to-fibroblast transition and proliferation [22]. Additionally, we have previously demonstrated that macrophage-derived PDFGD activates PDGFRβ, and inhibition of PDGFRβ effectively reduces the extent of fibrotic scarring after SCI [6]. Building upon our earlier findings, our current study reveals that inhibiting Galectin-3 blocks PDGFD-induced fibrosis in the injured and uninjured spinal cords. This suggests the active mediation of Galectin-3 in the formation of fibrotic scarring within the PDGFD/PDGFRβ pathway. Despite potential subtype variations, our study provides a foundational understanding of the synergetic interaction between Galectin-3 and PDGF. Additional data reveal that macrophage-derived Galectin-3 can be internalized by PDGFRβ^+^ fibroblasts in vitro, and its blockage results in reduced PDGFRβ expression. Together, our results indicate that Galectin-3-mediated PDGFRβ internalization contributes significantly to PDGF-induced fibrosis. In addition to the conventional activation pathway involving ligand-induced PDGFRβ dimerization and subsequent activation of downstream signaling cascades, our study proposes that Galectin-3 may augment and prolong activation of this pathway by facilitating PDGFRβ endocytosis, thereby affecting the progression of fibrosis. It is noteworthy that the regulation of fibrotic scar formation by Galectin-3 after SCI is a complex process involving various mechanisms. These mechanisms include inflammatory responses driven by macrophages and microglia, fibroblast proliferation, migration, and activation, as well as the potential role of astrocytes in compaction of the injury site. Our study provides preliminary evidence that Galectin-3 internalization by PDGFRβ^+^ fibroblasts enhance PDGFRβ expression, highlighting the direct role of Galectin-3 in promoting fibrosis after SCI.

In light of TD139’s promotion of motor function recovery, our study highlights the therapeutic potential of Galectin-3 inhibitors in the clinical management of patients after SCI. The high affinity inhibition of Galectin-3’s carbohydrate binding domain signifies the precise targeting achieved by TD139. However, substantiating evidence is warranted to establish the indispensability of this carbohydrate binding domain for inhibiting the role of Galectin-3. Future research could focus on constructing a carbohydrate-binding-deficient Galectin-3 mutant, such as Galectin-3-R186S, to assess its impact on fibrosis. Currently, electron microscope tomography in vitro has achieved the detection of Galectin-3 internalization via the CLICs pathway. The difficulty in detecting Galectin-3 internalization in vivo underscores the need for targeted antibody development. Furthermore, the question of whether Galectin-3 mediates the activation of PDGFRβ receptor phosphorylation and its downstream mechanisms remains to be thoroughly explored. Addressing these gaps in knowledge will significantly advance our understanding of the intricate molecular interactions underlying the observed phenomena.

## Conclusion

**Fig. 9:**
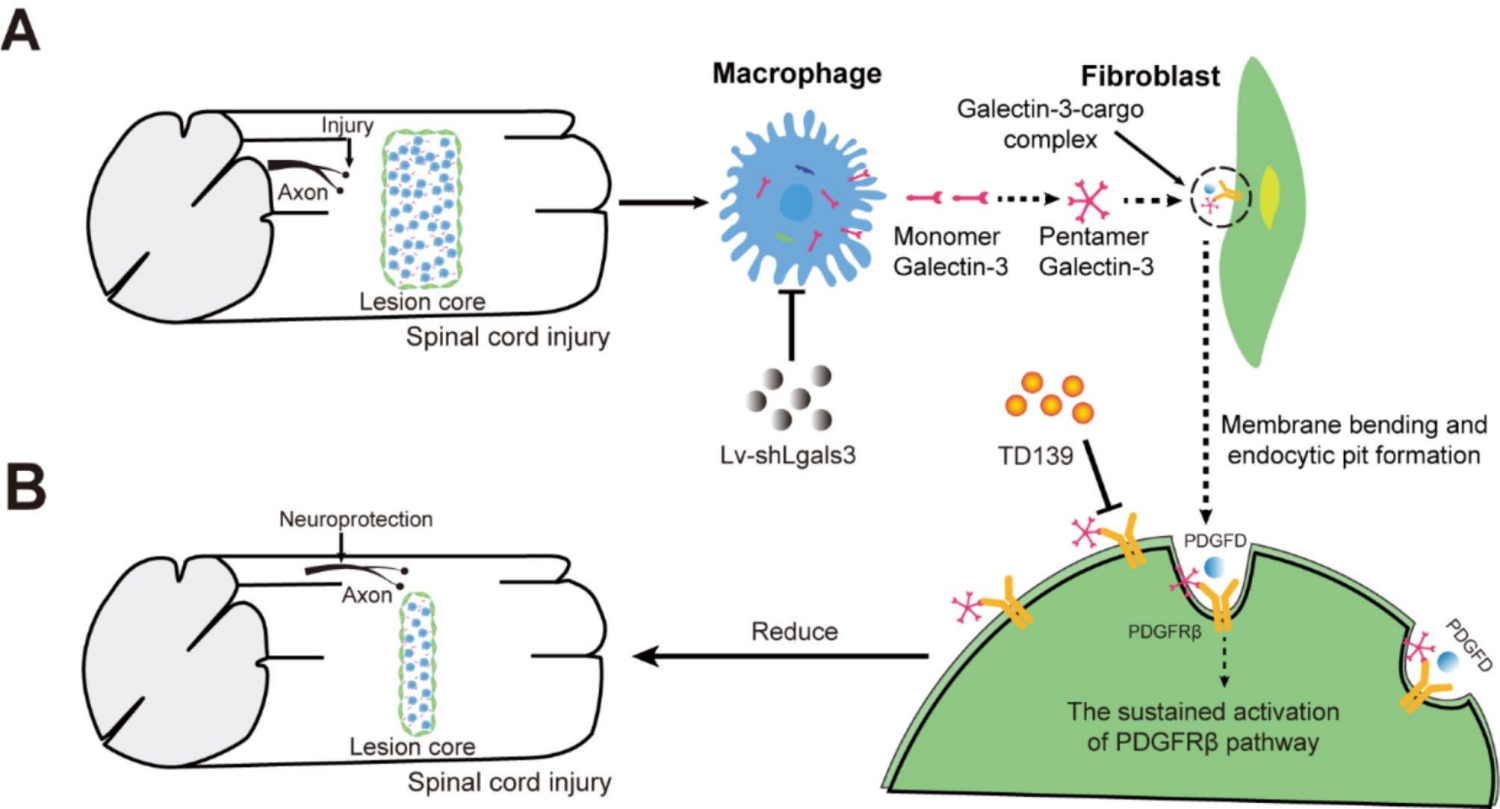
Model of Galectin-3 modulation of fibrotic scar formation at the lesion core after SCI. **A** Macrophages infiltrate the injury core after SCI, and macrophage-derived Galectin-3 may be involved in mediating the sustained activation of PDGFRβ receptors localized on the surface of fibroblasts and regulating the internalization of PDGFRβ [21, 32, 34]. Monomeric Galectin-3 recruited to the membrane is assembled into a pentameric-cargo complex, which affects membrane bending and endocytosis pit formation, thereby influencing the ligand-induced PDGFRβ endocytosis process. **B** Intrathecal injection of the pharmacological Galectin-3 inhibitor TD139 or in situ injection of Lv-shLgals3 reduced PDGFRβ^+^ fibrosis, promoted neuroprotection and motor function recovery after SCI

In summary, our study proposes a novel mechanistic insight into the interplay between macrophages and fibroblasts, elucidating Galectin-3’s influence on the formation of fibrotic scar at the injury core after SCI. The targeted inhibition Galectin-3 by TD139 or Lv-shLgals3 has demonstrated a significant reduction in fibrotic scar formation, concurrently fostering neuroprotection and facilitating motor function recovery post-SCI. Our in vitro experiments reveal the internalization of Galectin-3 into fibroblasts, leading to the upregulation of PDGFRβ expression. This suggests macrophage-derived Galectin-3 may play a potential role in mediating sustained internalized activation of PDGFRβ within fibroblasts (as depicted in Fig. 9). These findings lay the foundation for a deeper exploration of Galectin-3’ role in fibrotic scarring after SCI and offer potential directions for therapeutic interventions.

## Electronic supplementary material

Below is the link to the electronic supplementary material.

Supplementary Material 1

## Data Availability

The data used and/or analyzed during the current study are available from the corresponding author upon reasonable request.
