# Supplementary figures and images for: Galectin-3 inhibition reduces fibrotic scarring and promotes functional recovery after spinal cord injury in mice
Source: Cell Biosci. 2024 Oct 15;14:128. doi: 10.1186/s13578-024-01310-9 (PMC11481377; doi:10.1186/s13578-024-01310-9)

**Supplementary Figures**

**Figure S1**


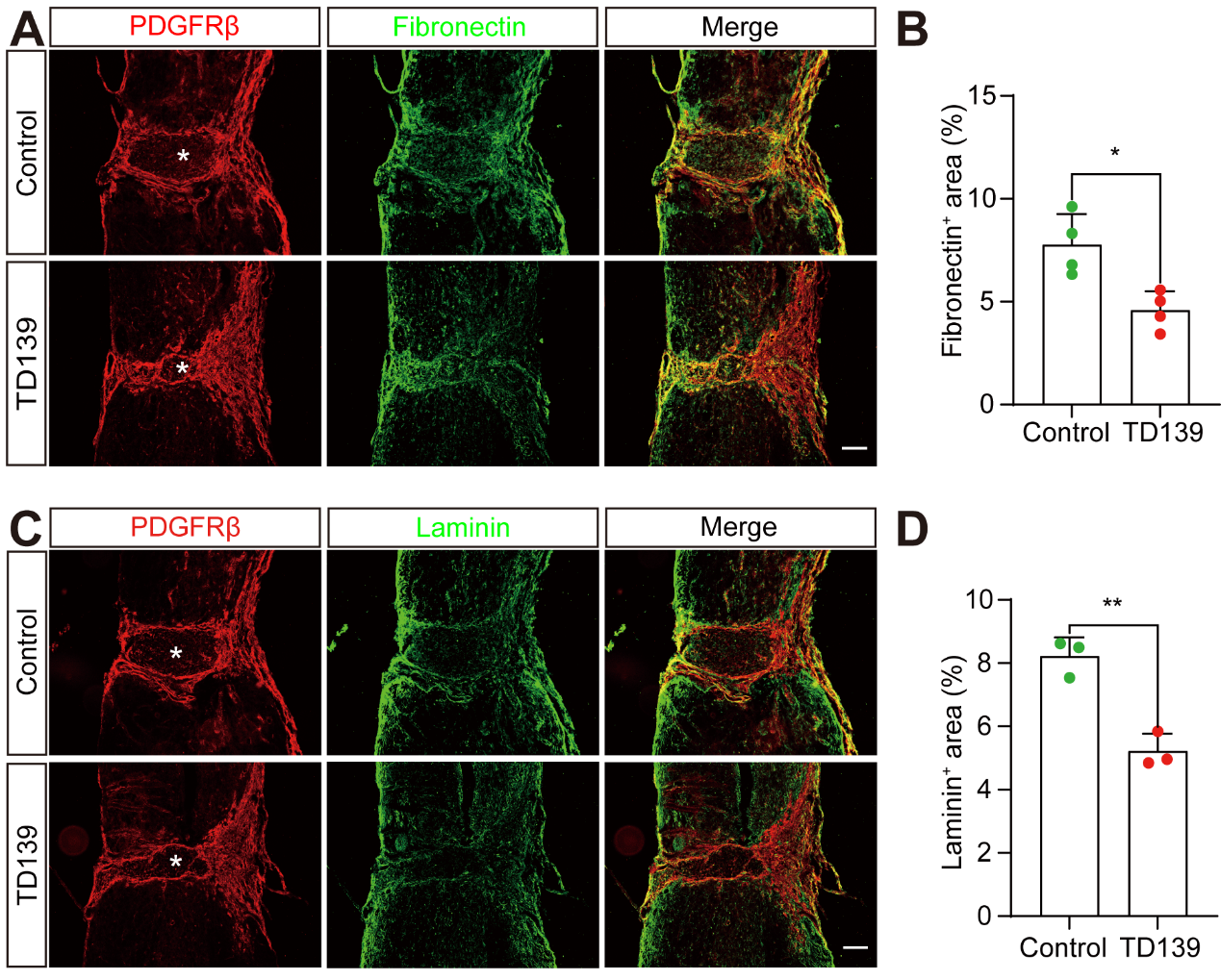


**Revised Figure S2**


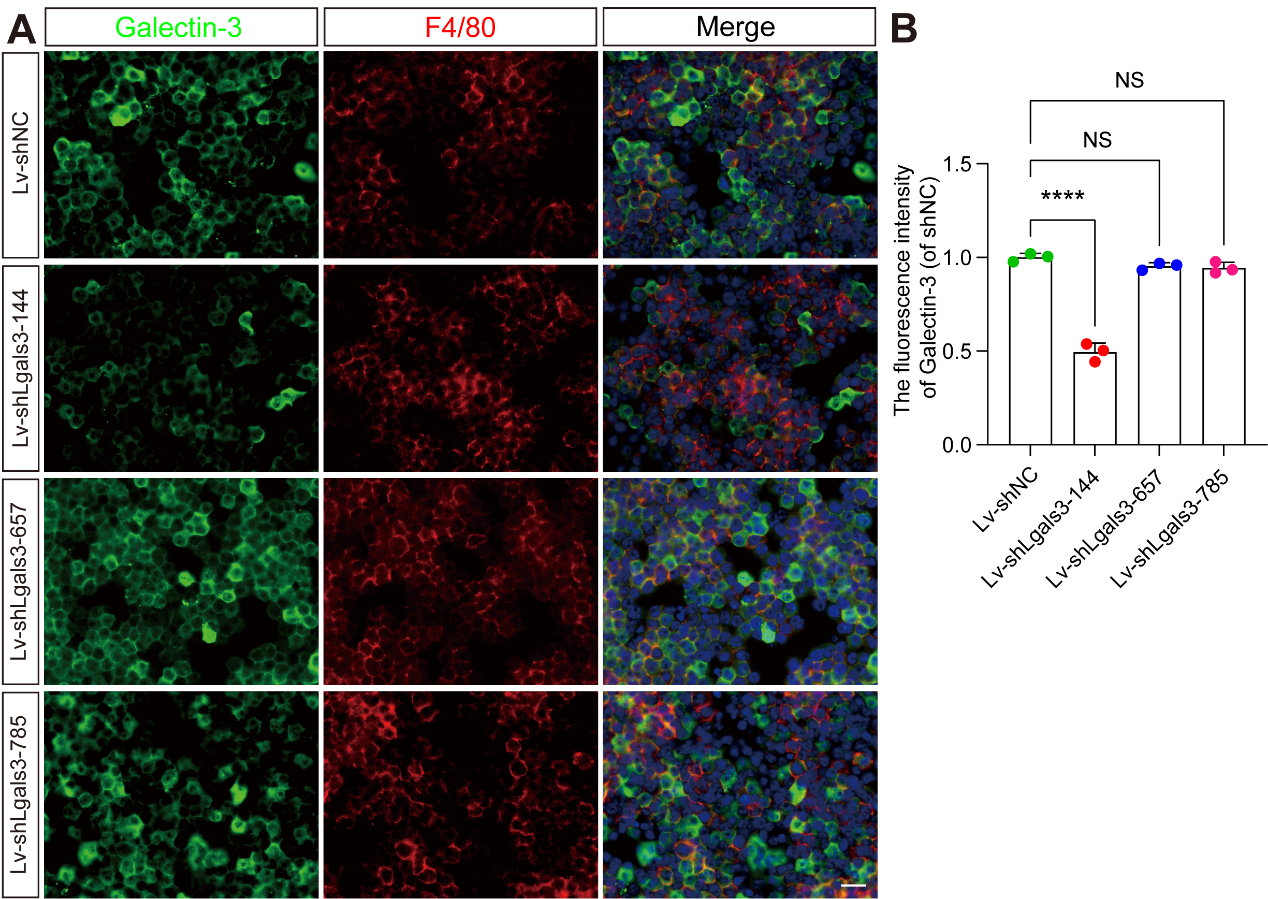


**Revised Figure S3**


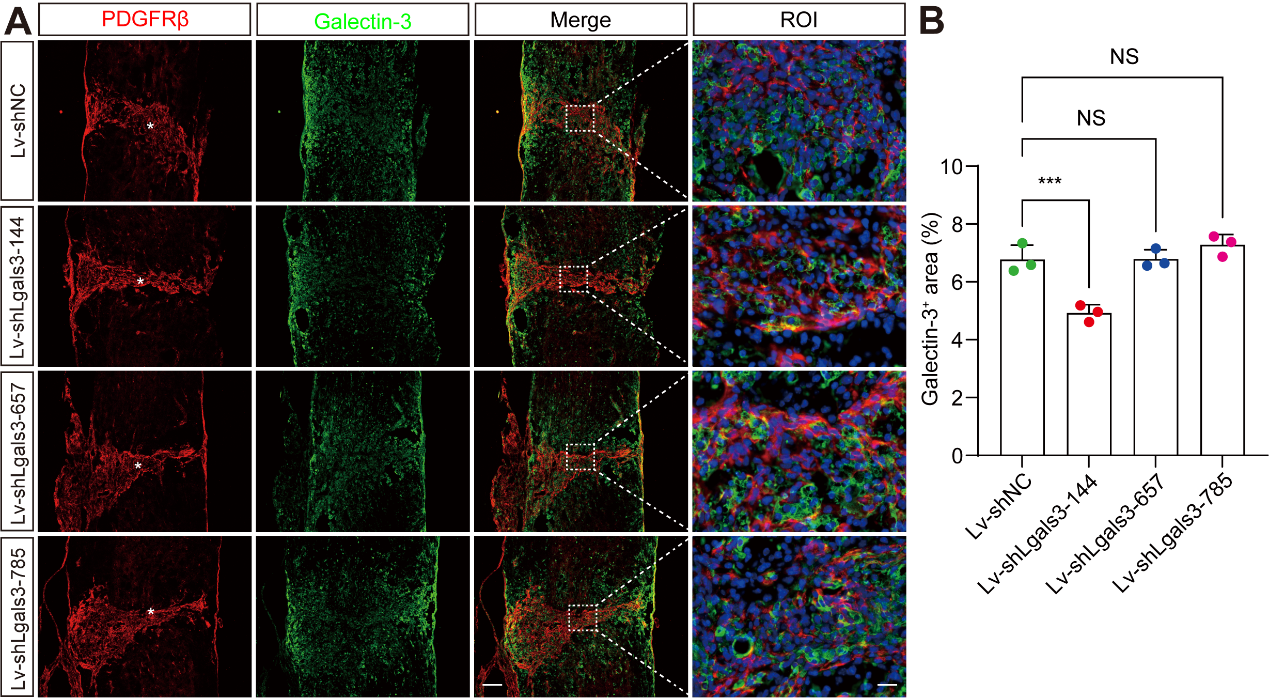


**Figure S4**


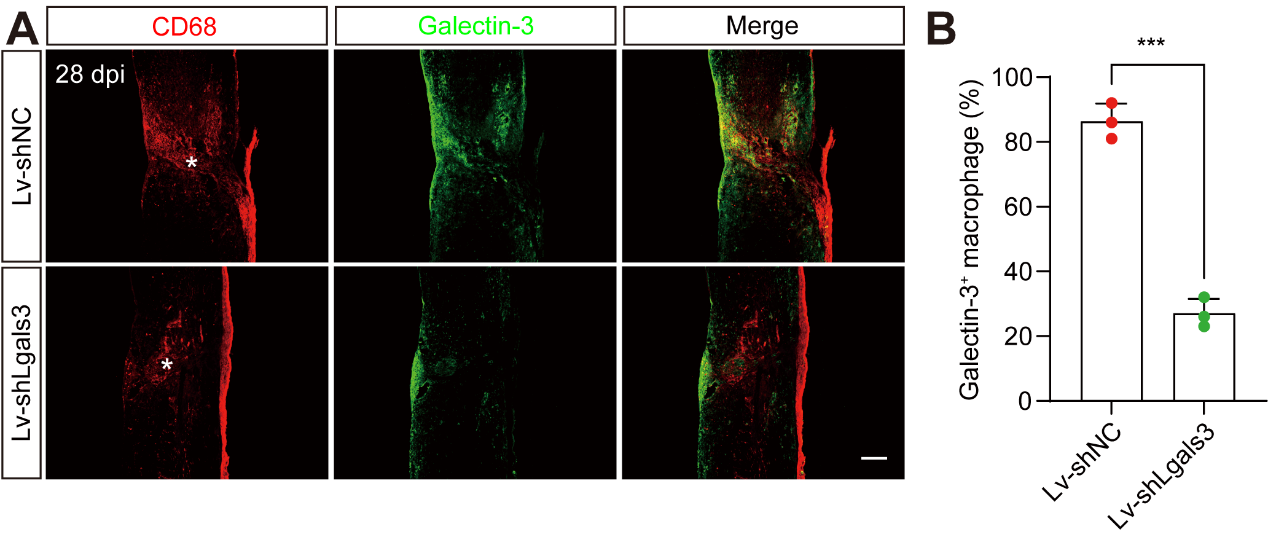

Supplement: Supplementary file 1 — Supplementary Material 1 [file 13578_2024_1310_MOESM1_ESM.docx]
